# Molecular hybrids of trivacant lacunary polyoxomolybdate and multidentate organic ligands†

**DOI:** 10.1039/d3sc03713d

**Published:** 2023-09-07

**Authors:** Atsuhiro Jimbo, Chifeng Li, Kentaro Yonesato, Tomoki Ushiyama, Kazuya Yamaguchi, Kosuke Suzuki

**Affiliations:** a Department of Applied Chemistry, School of Engineering, The University of Tokyo 7-3-1 Hongo, Bunkyo-ku Tokyo 113-8656 Japan ksuzuki@appchem.t.u-tokyo.ac.jp; b NIPPON STEEL Eco-Tech Corporation 2-1-38 Shiohama Kisarazu Chiba 292-0838 Japan

## Abstract

Functional molecular inorganic–organic hybrids of lacunary polyoxometalates and organic ligands attract much attention for advanced material applications. However, the inherent instability of lacunary polyoxomolybdates hinders the synthesis of hybrids and their utilization. Herein, we present a viable approach for the synthesis of molecular hybrids of trivacant lacunary Keggin-type polyoxomolybdates and multidentate organic ligands including carboxylates and phosphonates, which is based on the use of a lacunary structure stabilized by removable pyridyl ligands as a starting material.

## Abstract 2

This work presents a method for synthesizing hybrids of trivacant lacunary polyoxomolybdates [PMo_9_O_34_]^9−^ and multidentate organic ligands, including carboxylates and phosphonates, by using a lacunary species stabilized with removable pyridyl ligands.

## Graphical abstract

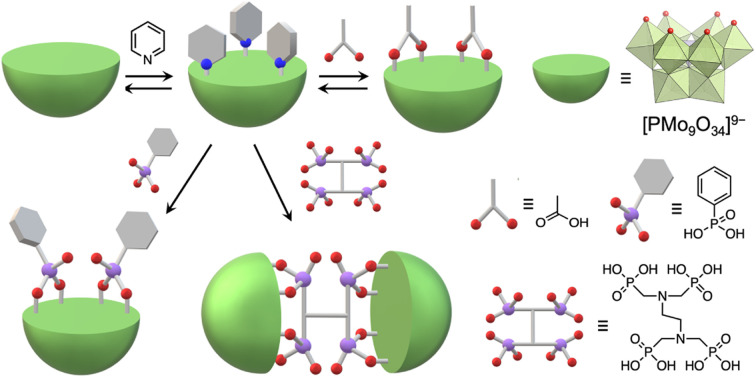

## Introduction

Inorganic–organic hybrids have received considerable interest owing to their diverse structures and properties, which render them attractive for various applications, including catalysis, photocatalysis, energy conversion, molecular recognition, and separation.^1^ These hybrids exhibit unique chemical and physical properties that arise from the synergistic effects of their components. For the construction of functional inorganic–organic hybrids, polyoxometalates (POMs), a class of structurally well-defined anionic metal oxide clusters (*e.g.*, W^6+^, Mo^6+^, and V^5+^) with diverse structures and constituent elements,^2^ stand out as attractive building units with distinctive properties such as redox, acid–base, and optical properties.^3^ In particular, lacunary POMs, which are obtained by removing several {MO_*x*_} units from parent POM structures, hold great potential for developing hybrid materials with diverse structures and properties owing to their structural versatility and wide range of bonding sites, directions, and constituent elements and the presence of highly reactive O atoms on the vacant sites for the introduction of various organic ligands. Although considerable efforts have been devoted to developing POM–organic hybrids by introducing organic ligands such as phosphates, silicates, alkoxides, and organometals into the vacant sites of lacunary POMs,^3,4^ successful results are restricted to lacunary polyoxotungstates, with only one report on the use of a monovacant lacunary polyoxomolybdate.^5^ This limitation arises from the inherent instability of polyoxomolybdates, which are prone to undesired structural transformation and decomposition during the reaction with organic ligands. In particular, lacunary polyoxomolybdates with multiple vacant sites, such as divacant [PMo_10_O_36_]^7−^ and trivacant [PMo_9_O_34_]^9−^, possess multiple reactive sites for organic ligands but are remarkably unstable,^6^ preventing the construction of polyoxomolybdate–organic hybrids.^3*c*^ Considering that polyoxomolybdates exhibit enhanced oxidative redox properties and higher stability of multielectron-reduced species compared to polyoxotungstates, the development of synthetic methods for polyoxomolybdate–organic hybrids using multivacant lacunary species is highly desirable for advanced materials with applications in various fields, including catalysis, photocatalysis, energy storage, and electronics.^7^

Recently, we developed a practical method to stabilize multivacant lacunary Keggin-type phosphomolybdates, namely trivacant [PMo_9_O_34_]^9−^ and divacant [PMo_10_O_36_]^7−^, by coordinating pyridine ligands to Mo atoms at the vacant sites.^8^ This reversible coordination through a monodentate Mo–N bond enables the use of the resulting pyridine-protected lacunary phosphomolybdates as precursors of POM–organic hybrids and the introduction of metal ions.^8,9^ However, the conjugation of organic ligands to lacunary polyoxomolybdates is currently limited to pyridine ligands. We envisaged that multidentate ligands such as carboxylates and phosphonates could provide more robust coordination to lacunary polyoxomolybdates than monodentate pyridine ligands, thus facilitating the formation of polyoxomolybdate–organic hybrids to explore their properties and applications.

**Fig. 1:**
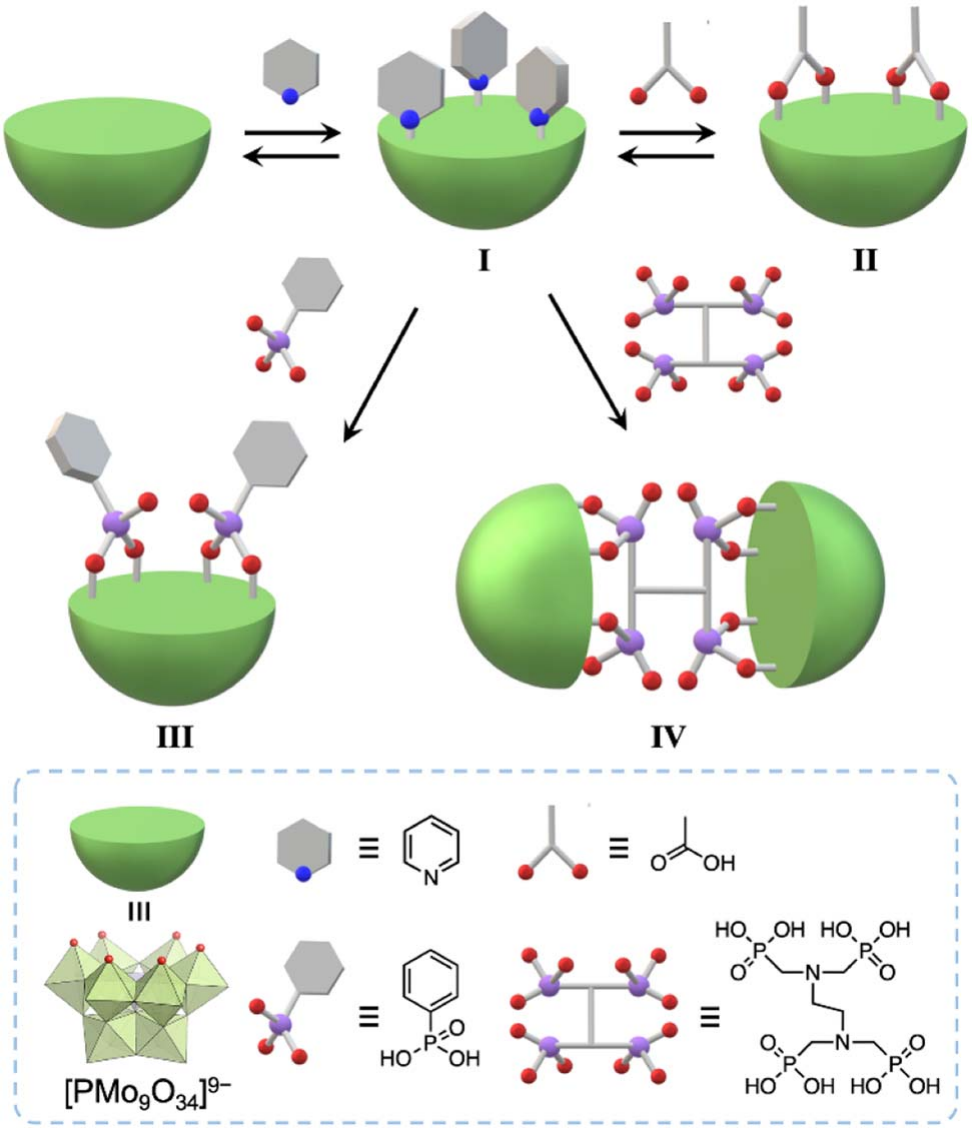
Schematic of the synthesis of molecular hybrids II, III, and IV, which consist of trivacant lacunary polyoxomolybdates and multidentate organic ligands, using pyridyl-protected I in organic solvents.

Herein, we developed a useful method to synthesize molecular hybrids of multivacant lacunary polyoxomolybdates and multidentate carboxylate and phosphonate ligands. By reacting pyridine-protected trivacant lacunary polyoxomolybdate I (TBA_3_[A-α-PMo_9_O_31_(C_5_H_5_N)_3_]; TBA = tetra-*n*-butylammonium, C_5_H_5_N = pyridine)^8*a*^ with multidentate organic ligands, *i.e.*, acetic acid, phenylphosphonic acid, and ethylenediamine-*N*,*N*,*N*′,*N*′-tetra(methylenephosphonic acid) (EDTMP), in organic solvents, we successfully synthesized monomer hybrids II and III and a dimer hybrid IV (Fig. 1).

## Results and discussion

**Fig. 2:**
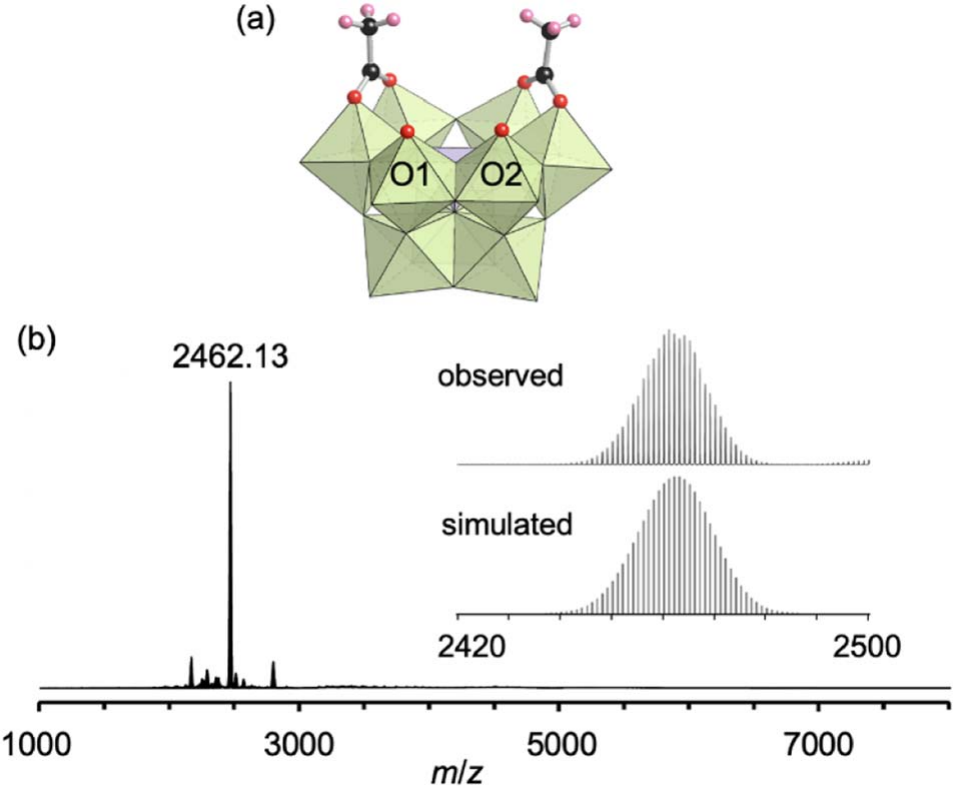
(a) Crystal structure of the anion part of II. (b) ESI mass spectrum of II in acetonitrile. Inset: enlarged spectrum (top) and simulated pattern for [TBA_4_(PMo_9_O_30_)(CH_3_COO)_2_]^+^ (*m*/*z*: 2462.14, bottom). The light green and light purple polyhedra represent [MoO_6_] and [PO_4_], respectively. The red, black, and pink spheres represent O, C, and H atoms, respectively.

First, we investigated the conjugation of carboxylate ligands to trivacant lacunary phosphomolybdate [PMo_9_O_34_]^9−^ by reacting pyridyl-protected I with an excess amount of acetic anhydride (see ESI† for details). The cold-spray ionization (CSI) mass spectrum of the reaction solution exhibited a set of signals centered at *m*/*z* 2462.1, which can be assigned to [TBA_4_(PMo_9_O_30_)(CH_3_COO)_2_]^+^ (theoretical *m*/*z*: 2462.5, Fig. S1a†), indicating the formation of II as a hybrid composed of a {PMo_9_} unit and two acetate ligands. The ^31^P NMR spectrum of the reaction solution showed a major signal at −3.9 ppm, suggesting that II was formed in high purity (Fig. S1b†). By adding diethyl ether to the reaction solution, single crystals of II suitable for an X-ray crystallographic analysis were obtained. In contrast, when I was reacted with a stoichiometric amount of acetic anhydride in 1,2-dichloroethane, the conjugation reaction hardly proceeded (Fig. S2†). The X-ray crystallographic analysis of II revealed a monomer structure containing two acetate ligands coordinated to Mo atoms at the vacant sites of [PMo_9_O_34_]^9−^*via* bidentate coordination (Fig. 2a and Table S1†). The adjacent two O atoms (O1 and O2) at the vacant sites remained unreacted as terminal oxo ligands (Mo=O), as indicated by their bond valence sum (BVS) values (1.69 and 1.81, Table S2†). The distances between the two O atoms of the coordinated acetate ligands (2.27 and 2.28 Å) became much shorter than those between the O and N atoms in I (2.91–2.96 Å), whereas the distance between the two O atoms (O1 and O2) at the vacant sites became longer (3.11 Å, Fig. S3†). These results show that the conjugation of the bidentate ligands induced structural distortion.

The electrospray ionization (ESI) mass spectrum of II in acetonitrile exhibited a signal set centered at *m*/*z* 2462.13, which was assignable to [TBA_4_(PMo_9_O_30_)(CH_3_COO)_2_]^+^ (theoretical *m*/*z*: 2462.14, Fig. 2b). In addition, the ^31^P NMR spectrum of II recorded immediately after dissolution in acetonitrile-*d*_3_ showed a single signal at −4.3 ppm (Fig. S4a†). Although these results revealed that the structure of II was preserved immediately after dissolution in acetonitrile, a ^31^P NMR spectrum of the solution recorded after five days showed the disappearance of the original signal at −4.3 ppm and the appearance of a new signal attributable to [PMo_12_O_40_]^3−^ at −2.7 ppm (Fig. S4b†). As reported in our previous paper, without protecting ligands, [PMo_9_O_34_]^9−^ easily undergoes structure transformation into a fully occupied plenary Keggin species [PMo_12_O_40_]^3−^.^8^ Therefore, this result is indicative of the gradual dissociation of the acetate ligands of II, causing the structural change of trivacant lacunary polyoxomolybdate to [PMo_12_O_40_]^3−^.

Taken together, the X-ray crystallographic analysis, ESI mass spectrometry, thermogravimetric (TG) analysis (Fig. S5†), and elemental analysis results revealed that the formula of II was TBA_3_[A-α-PMo_9_O_30_(CH_3_COO)_2_](H_2_O).

Next, we aimed to strengthen the conjugation to the trivacant lacunary polyoxomolybdate by employing phosphonate ligands, which exhibit higher hydrolytic stability than carboxylate ligands.^10^ The CSI mass spectrum of the reaction of I and phenylphosphonic acid (2 equivalents with respect to I) in nitromethane at room temperature for 2 h exhibited a set of signals centered at *m*/*z* 2658.4 that was assignable to [TBA_4_H_2_(PMo_9_O_30_)(C_6_H_5_PO_3_)_2_]^+^ (theoretical *m*/*z*: 2658.6, Fig. S6a†), indicating the formation of III as a hybrid comprising [PMo_9_O_34_]^9−^ and two phenylphosphonate ligands. The ^31^P NMR spectrum of the reaction solution exhibited two major signals at −4.1 and 17.0 ppm with an integration ratio of 1 : 2, which can be assigned to the P atom of the {PMo_9_} unit and those of the coordinated phenylphosphonates, respectively (Fig. S6b†). These results confirmed the quantitative conjugation of two phenylphosphonate ligands to the trivacant lacunary polyoxomolybdate, which is in sharp contrast with the conjugation of acetate ligands requiring an excess amount of ligand during the synthesis, as mentioned above.

**Fig. 3:**
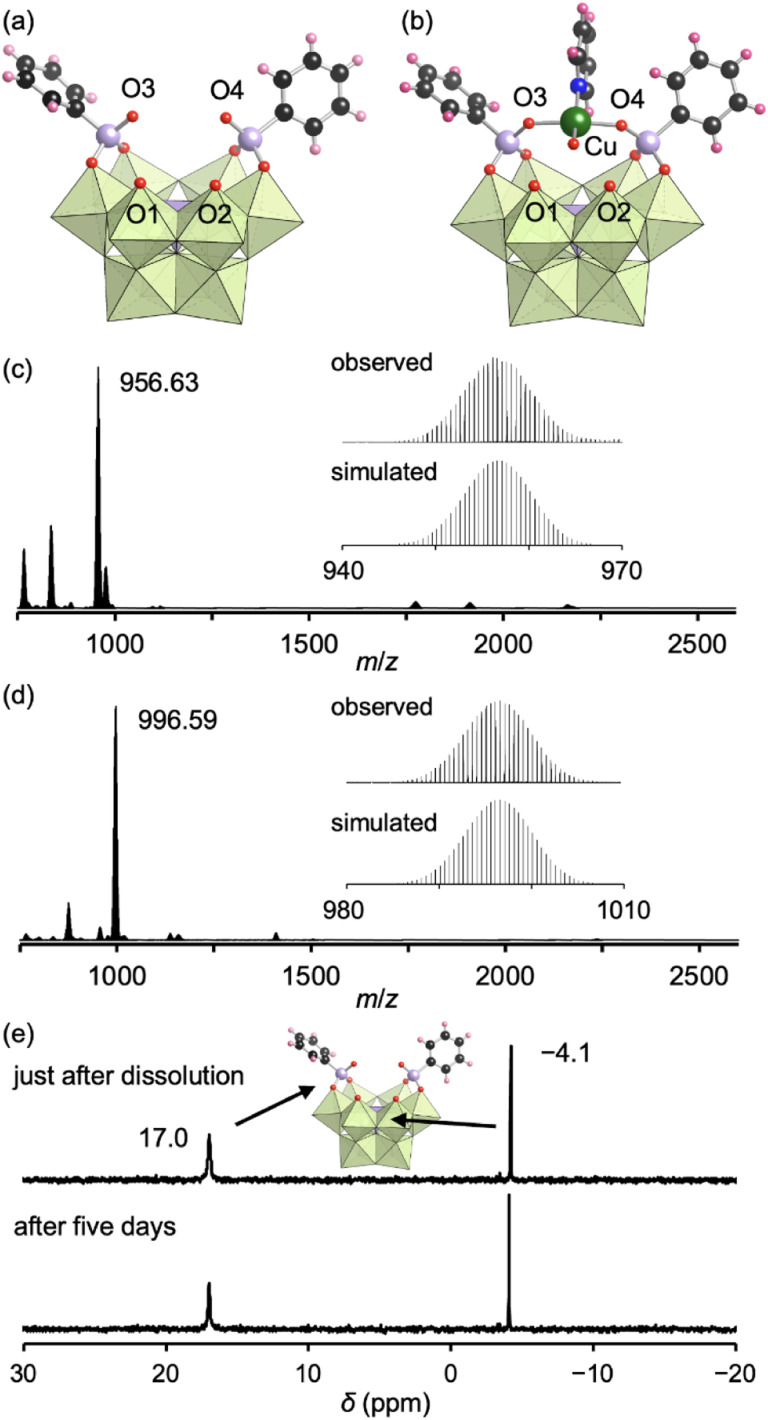
(a and b) Crystal structures of the anion parts of III (a) and III_Cu_ (b). (c) ESI mass spectrum of III in acetonitrile. Inset: enlarged spectrum (top) and simulated pattern for [TBA(PMo_9_O_29_)(C_6_H_5_PO_3_)_2_]^2−^ (*m*/*z*: 956.61, bottom). (d) ESI mass spectrum of III_Cu_ in acetonitrile. Inset: enlarged spectrum (top) and simulated pattern for [TBA(PMo_9_O_30_)(C_6_H_5_PO_3_)_2_Cu]^2−^ (*m*/*z*: 996.59, bottom). (e) ^31^P NMR spectra of III just after dissolution and after five days in acetonitrile-*d*_3_. The light green and light purple polyhedra represent [MoO_6_] and [PO_4_], respectively. The red, black, pink, blue, green, and light purple spheres represent O, C, H, N, Cu, and P atoms, respectively.

The addition of tetrahydrofuran to the reaction solution allowed obtaining single crystals of III suitable for an X-ray crystallographic analysis, which revealed a monomer structure bearing two bidentate phenylphosphonate ligands coordinated to Mo atoms at the vacant sites (Fig. 3a and Table S1†). The adjacent two O atoms at the vacant sites remained unreacted (O1 and O2) as terminal oxo ligands (Mo=O), as indicated by their BVS values (1.65 and 1.72) (Table S3†). The distances between the two O atoms of the coordinated phenylphosphonates in III (2.57 and 2.57 Å) were longer than those between the two O atoms of the coordinated acetate in II (2.27 and 2.28 Å, Fig. S3†) but shorter than those between the O and N atoms in I (2.91–2.96 Å, Fig. S3†). This anion structure of III resembles that of a previously reported phosphonate-conjugated polyoxotungstate.^4*a*^ The ESI mass spectrum of III in acetonitrile exhibited a signal set centered at *m*/*z* 956.63 assignable to [TBA(PMo_9_O_29_)(C_6_H_5_PO_3_)_2_]^2−^ (theoretical *m*/*z*: 956.61, Fig. 3c). The ^31^P NMR spectrum of III in acetonitrile-*d*_3_, which showed two signals at −4.1 and 17.0 ppm, remained unchanged at least for five days (Fig. 3e), revealing the considerably higher stability of III compared with that of I and II.^8*a*^ According to the X-ray crystallographic analysis, ESI mass spectrum, TG analysis (Fig. S7†), and elemental analysis, the formula of III was determined to be TBA_3_H_2_[A-α-PMo_9_O_30_(C_6_H_5_PO_3_)_2_](H_2_O)(C_5_H_5_N)_0.5_(CH_3_NO_2_)_0.5_.

Even when adding an excess amount of phenylphosphonic acid during the synthesis, the maximum number of conjugated ligands was two and the vacant site remained as Mo=O. Density functional theory calculations revealed that the standard Gibbs energy of reaction (Δ_r_*G*°) for the conjugation of two phenylphosphonate ligands to the trivacant lacunary polyoxomolybdate to form III ([PMo_9_O_31_(OH_2_)_3_]^3−^ + 2C_6_H_5_PO(OH)_2_ → H_2_[PMo_9_O_30_(C_6_H_5_PO_3_)_2_]^5−^ + 4H_2_O) was −37.1 kcal mol^−1^. In contrast, the Δ_r_*G*° for the conjugation of an additional phenylphosphonate ligand to the vacant site of III (H_2_[PMo_9_O_30_(C_6_H_5_PO_3_)_2_]^5−^ + C_6_H_5_PO(OH)_2_ → [PMo_9_O_28_(C_6_H_5_PO_3_)_3_]^3−^ + 2H_2_O) was +12.0 kcal mol^−1^. These results indicate that the formation of a product with three phenylphosphonate ligands is thermodynamically unfavorable, likely due to the structural distortion caused by the conjugation of an additional ligand.

Since III is stable in solution and possesses available O atoms on the vacant sites (O1 and O2) and phosphonate ligands (O3 and O4) that could act as coordination sites (Fig. 3a), we investigated the introduction of metal ions into III. By reacting III and Cu(ii) acetate (1 equivalent) in nitromethane, followed by the addition of diethyl ether, single crystals of III_Cu_ were obtained. The corresponding X-ray crystallographic analysis revealed that III_Cu_ contained a Cu(ii) ion coordinated by two O atoms of phenylphosphonates (O3 and O4), one water molecule, and one pyridine molecule in a square-planar coordination environment (Fig. 3b and Table S1†). The ESI mass spectrum of III_Cu_ in acetonitrile exhibited a signal set centered at *m*/*z* 996.59, which was assignable to [TBA(PMo_9_O_30_)(C_6_H_5_PO_3_)_2_Cu]^2−^ (theoretical *m*/*z*: 996.59, Fig. 3d). The results of the X-ray crystallographic analysis, ESI mass spectrum, TG analysis (Fig. S8†), and elemental analysis revealed that the formula of III_Cu_ was TBA_3_[A-α-PMo_9_O_30_(C_6_H_5_PO_3_)_2_Cu(C_5_H_5_N)(H_2_O)]. The two O atoms at the vacant sites (O1 and O2) in III remained unreacted as terminal oxo ligands (Mo=O), as indicated by their BVS values (1.63 and 1.67, Table S4†). This observation is consistent with the previously reported introduction of a metal ion into a phosphonate-containing polyoxotungstate.^11^ Notably, no additional Cu(ii) ions could be introduced by reacting III_Cu_ with an excess amount of Cu(ii) acetate.

In our previous synthesis of Mn-containing {PMo_9_} polyoxomolybdates,^9*a*^ the pyridine ligands of I were removed upon metal introduction. In contrast, the introduction of the Cu(ii) ion into III did not displace the phosphonate ligands, which served as metal coordination sites. These results confirm the stronger coordination of phosphonate ligands to polyoxomolybdates compared with pyridine ligands, which could be exploited to achieve enhanced structural and physical property diversity through the design of polyoxomolybdates bearing organophosphate ligands and additional metal ions. In fact, we synthesized a series of analogous structures to III_Cu_ by reacting III with metal ions such as Co^2+^, Ni^2+^, {VO}^2+^, and Ag^+^ instead of Cu^2+^ (Fig. S9†).

**Fig. 4:**
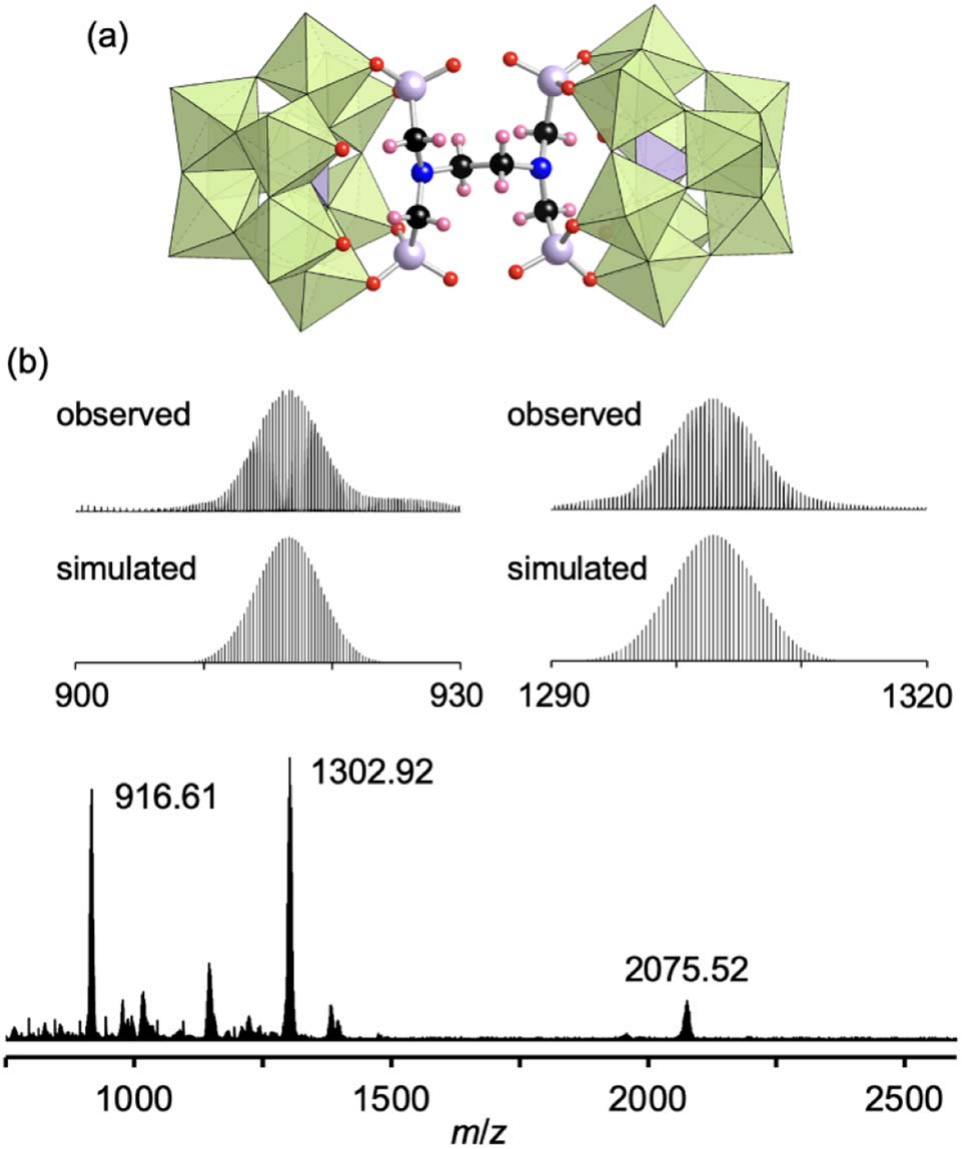
(a) Crystal structure of the anion part of IV. (b) ESI mass spectrum of IV in acetonitrile. Inset: enlarged spectra (top) and simulated patterns for [TBA_2_(PMo_9_O_30_)_2_(C_6_H_16_N_2_O_12_P_4_)]^4−^ (*m*/*z*: 916.62, bottom left) and [TBA_3_(PMo_9_O_30_)_2_(C_6_H_16_N_2_O_12_P_4_)]^3−^ (*m*/*z*: 1302.92, bottom right). The light green and light purple polyhedra represent [MoO_6_] and [PO_4_], respectively. The red, black, pink, blue, and light purple spheres represent O, C, H, N, and P atoms, respectively.

Finally, by utilizing the robust conjugation of phosphonate ligands to the lacunary polyoxomolybdates, we investigated the synthesis of stable oligomeric structures with a polyfunctionalized phosphonate ligand, *i.e.*, EDTMP. The reaction of I and EDTMP (1 equivalent) was performed in *N*,*N*-dimethylformamide at 80 °C. The CSI mass spectrum and ^31^P NMR spectrum of the reaction solution indicated the formation of a dimer structure IV comprising two [PMo_9_O_34_]^9−^ units and one EDTMP ligand (Fig. S10†). The X-ray crystallographic analysis of IV revealed that the anion exhibited a dimer structure with EDTMP acting as a tetradentate ligand coordinated to two [PMo_9_O_34_]^9−^ units (Fig. 4a and Table S1†). The BVS values of the adjacent two O atoms at the vacant sites of each [PMo_9_O_34_]^9−^ unit (1.72–1.79, Table S5†) indicated that they remained unreacted as terminal oxo ligands (Mo=O). The ESI mass spectrum of IV in acetonitrile exhibited a series of signal sets centered at *m*/*z* = 916.61, 1302.92, and 2075.52, which were assignable to [TBA_2_H_4_(PMo_9_O_30_)_2_(C_6_H_12_N_2_O_12_P_4_)]^4−^ (theoretical *m*/*z*: 916.62), [TBA_3_H_4_(PMo_9_O_30_)_2_(C_6_H_12_N_2_O_12_P_4_)]^3−^ (theoretical *m*/*z*: 1302.92), and [TBA_4_H_4_(PMo_9_O_30_)_2_(C_6_H_12_N_2_O_12_P_4_)]^2−^ (theoretical *m*/*z*: 2075.53), respectively (Fig. 4b), confirming the retention of the hybrid structure of IV in solution. On the basis of the X-ray crystallographic analysis, ESI mass spectrometry, TG analysis (Fig. S11†), and elemental analysis results, the formula of IV was determined to be TBA_6_H_4_[(A-α-PMo_9_O_30_)_2_(C_6_H_12_N_2_(PO_3_)_4_)].

## Conclusions

In conclusion, we successfully synthesized POM–organic molecular hybrids II and III by conjugating multidentate phosphonate and carboxylate ligands to a trivacant lacunary Keggin-type polyoxomolybdate using a pyridyl-protected species as a starting material. The phosphonate ligands coordinate more strongly to the vacant sites of the lacunary polyoxomolybdate than pyridine and carboxylic acid ligands, enabling the introduction of foreign metal cations to afford III_Cu_ and the formation of a dimer structure IV. This synthetic approach holds promise for the preparation of molecular hybrids with unexplored structures and properties.

## Data availability

The data supporting this manuscript is available in the ESI† of and available on request.

## Author contributions

K. S. conceived and directed the project. A. J., C. L., T. U. performed the synthesis and characterizations. A. J. and K. Y. performed the crystallographic analysis. A. J. and K. S. performed the DFT calculations. All authors analyzed and discussed the results. A. J., K. Y., K. Y., K. S. co-wrote the manuscript.

## Conflicts of interest

There are no conflicts of interest to declare.

## Supplementary Material

SC-014-D3SC03713D-s001

SC-014-D3SC03713D-s002
