# Supplementary material for: Molecular hybrids of trivacant lacunary polyoxomolybdate and multidentate organic ligands
Source: Chem Sci. 2023 Sep 7;14(37):10280–4. doi: 10.1039/d3sc03713d (PMC10530144; doi:10.1039/d3sc03713d)
Supplement: SC-014-D3SC03713D-s001 [file SC-014-D3SC03713D-s001.pdf]

Electronic Supplementary Material (ESI) for Chemical Science.
This journal is © The Royal Society of Chemistry 2023

Electronic Supplementary Information (ESI) for

Molecular hybrids of trivacant lacunary polyoxomolybdate and multidentate organic ligands

Atsuhiro Jimbo,[a] Chifeng Li,[a] Kentaro Yonesato,[a] Tomoki Ushiyama,[b] Kazuya Yamaguchi[a] and Kosuke Suzuki*[a]

[a]*Department of Applied Chemistry, School of Engineering, The University of Tokyo, 7-3-1 Hongo, Bunkyo-ku, Tokyo 113-8656, Japan.*

[b]*NIPPON STEEL Eco-Tech Corp., 2-1-38 Shiohama, Kisarazu, Chiba 292-0838, Japan.*

Contents

	page
**Experimental Section**	S1–S3
**Table S1–S5**	S4–S8
**Fig. S1–S11**	S9–S16
**Additional references**	S16

Experimental Section

Instruments: Electrospray ionization mass (ESI mass) spectra were recorded on a Shimadzu LCMS-9050 instrument and Waters Xevo G2-XS QTof instrument. Cold-spray ionization mass (CSI mass) spectra were recorded on JEOL JMS-T100CS instrument. IR spectra were measured on Jasco FT/IR-4100 instrument using KCl disks. NMR spectra were recorded on a JEOL ECA-500 spectrometer (^{31}P, 202.47 MHz) using 5 mm tubes. Chemical shifts (δ) are reported in upfield from H_3PO_4 (solvent, D_2O) for ^{31}P NMR spectra. Thermogravimetric and differential thermal analyses (TG-DTA) were performed on Rigaku Thermo plus EVO2 TG-DTA 8122 instrument under N_2 atmosphere to check the number of solvent molecules from the weight loss values and the decomposition temperature. Inductively coupled plasma atomic emission spectroscopy (ICP-AES) analyses were performed on Shimadzu ICPS-8100 instrument. Elemental analyses for C, H, and N were performed on Elementar vario MICRO cube at the Elemental Analysis Centre of the School of Science of the University of Tokyo.

Materials: Acetonitrile, *N*,*N*-dimethylformamide (DMF), tetrahydrofuran (THF), 1,2-dichloroethane, diethyl ether, acetic anhydride, copper(II) acetate monohydrate, cobalt(II) acetylacetonate dihydrate, and nickel(II) acetylacetonate dihydrate were purchased from Kanto Chemical. Nitromethane, phenylphosphonic acid, ethylenediamine-*N*,*N*,*N′*,*N′*-tetra(methylenephosphonic acid) (EDTMP), and acetonitrile-d_3 were purchased from Tokyo Chemical Industry. Vanadyl acetylacetonate was purchased from Acros Organics. Silver acetate was purchased from Sigma Aldrich. **I** ($TBA_3[A\text{-}\alpha\text{-}PMo_9O_{31}(C_5H_5N)_3]$) was synthesized according to our previous

report.[S1]

Single-Crystal X-ray Diffraction Analysis: Diffraction measurements were performed on a Rigaku XtaLab Synergy-R diffractometer with rotating-anode Mo Kα radiation (λ = 0.71073 Å, 50 kV, 24 mA) at 93 K. The data were collected and processed using CrysAlisPro.[S2] In the reduction of data, Lorentz and polarization corrections were made. Structural analyses were performed using WinGX.[S3] All structures were solved by SHELXT-2018/2 and refined by SHELXL-2018/3.[S4] All non-hydrogen atoms were refined anisotropically. Highly disordered TBA ions and solvent molecules were omitted by using SQUEEZE program.[S5] CCDC-2282515, 2282516, 2282517, and 2282518 contain the supplementary crystallographic data for **II**, **III**, **III$_{Cu}$** and **IV**, respectively. These data can be obtained free of charge from The Cambridge Crystallographic Data Centre via www.ccdc.cam.ac.uk/data_request/cif.

Bond Valence Sum (BVS) Calculations: BVS values were calculated by the expression for the variation of the length r_{ij} of a bond between two atoms i and j in observed crystal with valence Vi:

$$V_i = \sum_j \exp\left(\frac{r'_0 - r_{ij}}{B}\right)$$

where B is constant equal to 0.37 Å, r'_0 is bond valence parameter for a given atom pair.[S6, S7]

Density Functional Theory (DFT) Calculations: Computational studies were performed at Research Center for Computational Science, Okazaki, Japan. All calculations were performed with Gaussian 16 Rev B.01 package. The geometry optimizations were performed using CAM-B3LYP functional with employing the moderate-size basis set, LANL2DZ ECP for Mo and 6-31G* for P, O, C, H. The frequency calculations were performed using CAM-B3LYP functional with employing the basis set, LANL2DZ ECP for Mo and 6-31+G** for P, O, C, H. The solvent effects (acetonitrile) were included using the polarizable continuum model (PCM) with scaled van der Waals radii. After the geometry optimizations, the standard Gibbs energy of reaction ($\Delta_r G°$) was obtained by frequency calculations.

Synthesis of TBA$_3$[A-α-PMo$_9$O$_{30}$(CH$_3$COO)$_2$] (II): To acetic anhydride (4.0 mL), **I** (80 mg) was added, and the resulting solution was stirred for 2 h at 0°C, followed by filtration through a membrane filter. After the addition of diethyl ether (2.0 mL), the filtrate was kept at 5°C. The yellow crystals of **II** suitable for X-ray crystallographic analysis were obtained after 3 day (20% yield). Elemental analysis calcd (%) for TBA$_3$[PMo$_9$O$_{30}$(CH$_3$COO)$_2$](H$_2$O): C, 27.91; H, 5.22; N, 1.88; P, 1.38; Mo, 38.59. Found: C, 28.05; H, 5.16; N, 2.11; P, 1.36; Mo, 39.29. Positive ion MS (ESI, acetonitrile): m/z 2462.13 (calcd. 2462.14 for [TBA$_4$(PMo$_9$O$_{30}$)(CH$_3$COO)$_2$]$^+$). IR (KCl pellet, cm^{-1}): 3417, 2962, 2936, 2874, 1635, 1525, 1483, 1380, 1347, 1151, 1065, 1014, 940, 919, 879, 814, 756, 618, 526, 419.

Synthesis of TBA$_3$H$_2$[A-α-PMo$_9$O$_{30}$(C$_6$H$_5$PO$_3$)$_2$] (III): To nitromethane (4.0 mL), phenylphosphonic acid (24 mg, 151 μmol) and **I** (176 mg, 75 μmol) were added, and the resulting solution was stirred for 2 h at room temperature (~25°C), followed by filtration through a membrane filter. After the addition of THF (5.0 mL), the filtrate was kept at 25°C. The pale-yellow crystals of **III** suitable for X-ray crystallographic analysis were obtained

after 1 day (24% yield). Elemental analysis calcd (%) for $TBA_3H_2[PMo_9O_{30}(C_6H_5PO_3)_2](H_2O)(C_5H_5N)_{0.5}(CH_3NO_2)_{0.5}$: C, 30.22; H, 5.07; N, 2.24; P, 3.71; Mo, 34.49. Found: C, 30.23; H, 4.82; N, 2.46, P, 3.65, Mo, 34.40. Negative ion MS (ESI, acetonitrile): *m/z* 956.63 (calcd. 956.61 for $[TBAH_2(PMo_9O_{30})(C_6H_5PO_3)_2]^{2-}$). IR (KCl pellet, cm^{-1}): 3448, 3066, 2962, 2935, 2874, 2739, 2364, 2341, 1892, 1636, 1539, 1485, 1438, 1381, 1254, 1139, 1088, 1017, 987, 943, 926, 910, 865, 755, 698, 607, 552.

Synthesis of $TBA_3[A\text{-}\alpha\text{-}PMo_9O_{30}(C_6H_5PO_3)_2Cu(C_5H_5N)(H_2O)]$ (III_{Cu}): To nitromethane (4.0 mL), $Cu(OAc)_2{\cdot}H_2O$ (11.7 mg, 83 μmol) and **III** (200 mg, 83 μmol) were added, and the resulting solution was stirred for 1 h at room temperature (~25°C), followed by filtration through a membrane filter. After addition of diethyl ether (1.0mL), the filtrate was kept at 25°C. The blue crystals of $\mathbf{III_{Cu}}$ suitable for X-ray crystallographic analysis were obtained after 1 day (20% yield). Elemental analysis calcd (%) for. $TBA_3[PMo_9O_{30}(C_6H_5PO_3)_2Cu(C_5H_5N)(H_2O)]$: C, 30.32; H, 4.89; N, 2.18; P, 3.61; Mo, 33.54. Found: C, 30.05; H, 4.93; N, 2.17; P, 3.48; Mo, 33.48. Negative ion MS (ESI, acetonitrile): *m/z* 996.59 (calcd. 996.59 for $[TBA(PMo_9O_{30})(C_6H_5PO_3)_2Cu]^{2-}$). IR (KCl pellet, cm^{-1}): 3441, 3076, 2962, 2935, 2874, 2736, 2363, 2341, 1895, 1627, 1540, 1484, 1438, 1381, 1254, 1139, 1077, 1020, 1007, 986, 943, 928, 908, 865, 742, 698, 595, 558. In a similar manner, introduction of Co^{2+}, Ni^{2+}, $\{VO\}^{2+}$, and Ag^+ ions into **III** was performed by the reaction of **III** with cobalt(II) acetylacetonate dihydrate, nickel(II) acetylacetonate dihydrate, vanadyl acetylacetonate, and silver acetate, respectively.

Synthesis of $TBA_6H_4[(A\text{-}\alpha\text{-}PMo_9O_{30})_2(C_6H_{12}N_2(PO_3)_4)]$ (IV): To DMF (4.0 mL), EDTMP (24 mg, 151 μmol) and **I** (176 mg, 75 μmol) were added, and the resulting solution was stirred for 2 h at 80°C, followed by filtration through a membrane filter. By addition of an excess amount of diethyl ether (~ 200 mL) to the reaction solution, light green powder of **IV** was obtained (78% yield). In contrast, by addition diethyl ether (1.0 mL) to the reaction solution, the pale-yellow crystals of **IV** suitable for X-ray crystallographic analysis were obtained after 1 day (4% yield). Elemental analysis calcd (%) for $TBA_6[(PMo_9O_{30})_2(C_6H_{12}N_2(PO_3)_4)]$: C, 26.43; H, 5.04; N, 2.42; P, 4.01; Mo, 37.26. Found: C, 26.05; H, 5.00; N, 2.77, P, 3.95, Mo, 37.57. Negative ion MS (ESI, acetonitrile): *m/z* 916.61 (calcd. 916.62 for $[TBA_2H_4(PMo_9O_{30})_2(C_6H_{12}N_2P_4O_{12})]^{4-}$), 1302.92 (calcd. 1302.92 for $[TBA_3H_4(PMo_9O_{30})_2(C_6H_{12}N_2P_4O_{12})]^{3-}$), 2075.52 (calcd. 2075.53 for $[TBA_4H_4(PMo_9O_{30})_2(C_6H_{12}N_2P_4O_{12})]^{2-}$). IR (KCl pellet, cm^{-1}): 3440, 3064, 2962, 2937, 2874, 1631, 1483, 1469, 1381, 1254, 1151, 1078, 1028, 946, 914, 867, 748, 606, 577, 518, 469, 450, 419.

Table S1 Crystallographic data of **II**, **III**, **III$_{Cu}$** and **IV**

	**II**	**III**	**III$_{Cu}$**	**IV**
Crystal system	Monoclinic	Monoclinic	Monoclinic	Triclinic
Space group	$P2_1/c$	Cc	Cc	$P\bar{1}$
a (Å)	26.5817(7)	27.5817(4)	27.4763(4)	14.2945(2)
b (Å)	14.4781(2)	21.2628(3)	21.2915(3)	24.5625(4)
c (Å)	24.2574(8)	17.2310(3)	17.3843(3)	29.1153(5)
α (deg)	90	90	90	111.6533(15)
β (deg)	115.367(3)	105.7192(18)	105.5757(15)	98.3822(14)
γ (deg)	90	90	90	95.1863(13)
Volume (Å^3)	8435.4(4)	9727.5(3)	9796.6(2)	9283.5(3)
Z	4	4	4	2
GOF	1.056	1.048	1.058	1.050
$R_1[I > 2\sigma(I)]$	0.0798	0.0465	0.0381	0.0775
wR_2	0.2115	0.1155	0.1024	0.2217

$R_1 = \Sigma||F_o|-|F_c||/\Sigma|F_o|$, $wR_2 = \{\Sigma[w(F_o^2 - F_c^2)]/\Sigma[w(F_o^2)^2]\}^{1/2}$.

Table S2 BVS values of **II**

Mo1	6.12	O9	1.89	O23	1.94
Mo2	6.11	O10	1.92	O24	1.89
Mo3	6.01	O11	2.01	O25	1.97
Mo4	6.09	O12	2.02	O26	1.78
Mo5	6.03	O13	1.96	O27	1.77
Mo6	5.97	O14	2.04	O28	1.75
Mo7	5.98	O15	1.97	O29	1.82
Mo8	5.93	O16	2.08	O30	1.74
Mo9	5.86	O17	2.01	O31	1.75
P1	4.79	O18	2.02	O32	1.82
**O1** [a]	**1.69**	O19	2.01	O33	1.80
**O2** [a]	**1.81**	O20	2.05	O34	1.77
O7	1.91	O21	1.79		
O8	1.88	O22	1.87		

[a] Oxygen atoms at the vacant site of lacunary polyoxomolybdate.

Table S3 BVS values of **III**

Mo1	6.11	O7	1.87	O23	1.87
Mo2	6.07	O8	1.81	O24	1.86
Mo3	6.10	O9	1.82	O25	1.94
Mo4	6.13	O10	1.82	O26	1.89
Mo5	5.99	O11	1.79	O27	1.95
Mo6	5.96	O12	1.92	O28	1.87
Mo7	6.04	O13	1.99	O29	1.78
Mo8	5.98	O14	2.00	O30	1.82
Mo9	6.05	O15	1.97	O31	1.79
P1	4.76	O16	2.07	O32	1.73
**O1** [a]	**1.65**	O17	1.98	O33	1.75
**O2** [a]	**1.72**	O18	2.05	O34	1.85
O3	1.28	O19	2.07	O35	1.77
O4	1.25	O20	2.00	O36	1.84
O5	1.88	O21	2.01		
O6	1.84	O22	2.03		

[a] Oxygen atoms at the vacant site of lacunary polyoxomolybdate.

Table S4 BVS values of $\mathbf{III_{Cu}}$

Mo1	6.13	O6	1.84	O22	2.08
Mo2	6.09	O7	1.81	O23	1.86
Mo3	6.03	O8	1.91	O24	1.87
Mo4	6.07	O9	1.82	O25	1.90
Mo5	5.98	O10	1.84	O26	1.92
Mo6	6.00	O11	1.86	O27	1.96
Mo7	6.08	O12	1.93	O28	1.79
Mo8	6.02	O13	1.95	O29	1.81
Mo9	6.01	O14	2.01	O30	1.79
Cu1	1.99	O15	1.99	O31	1.80
P1	4.79	O16	2.05	O32	1.72
**O1** [a]	**1.63**	O17	2.00	O33	1.75
**O2** [a]	**1.67**	O18	2.08	O34	1.83
O3	1.83	O19	2.06	O35	1.82
O4	1.76	O20	2.01	O36	1.81
O5	1.91	O21	1.99	O1W	0.42

[a] Oxygen atoms at the vacant site of lacunary polyoxomolybdate.

Table S5 BVS values of **IV**.

Mo11	6.02	O114	1.98	Mo21	6.11	O214	1.98
Mo12	6.10	O115	1.96	Mo22	6.05	O215	1.97
Mo13	6.12	O116	2.13	Mo23	6.10	O216	2.00
Mo14	6.10	O117	1.98	Mo24	6.00	O217	1.98
Mo15	6.10	O118	2.10	Mo25	5.92	O218	2.09
Mo16	6.17	O119	2.01	Mo26	6.12	O219	2.02
Mo17	5.97	O120	1.99	Mo27	6.05	O220	1.96
Mo18	6.03	O121	1.98	Mo28	5.97	O221	2.05
Mo19	6.05	O122	2.06	Mo29	6.05	O222	2.11
P1	4.88	O123	1.87	P2	4.86	O223	1.86
**O101** [a]	**1.79**	O124	1.88	**O201** [a]	**1.77**	O224	1.85
**O102** [a]	**1.79**	O125	1.94	**O202** [a]	**1.72**	O225	1.87
O103	1.25	O126	1.90	O203	1.30	O226	1.92
O104	1.26	O127	2.00	O204	1.23	O227	1.93
O105	1.85	O128	1.82	O205	1.86	O228	1.83
O106	1.81	O129	1.82	O206	1.80	O229	1.79
O107	1.83	O130	1.81	O207	1.86	O230	1.85
O108	1.82	O131	1.82	O208	1.86	O231	1.82
O109	1.85	O132	1.76	O209	1.84	O232	1.75
O110	1.85	O133	1.80	O210	1.82	O233	1.75
O111	1.86	O134	1.81	O211	2.83	O234	1.81
O112	1.97	O135	1.80	O212	1.99	O235	1.90
O113	1.98	O136	1.80	O213	1.99	O236	1.81

[a] Oxygen atoms at the vacant site of lacunary polyoxomolybdate.

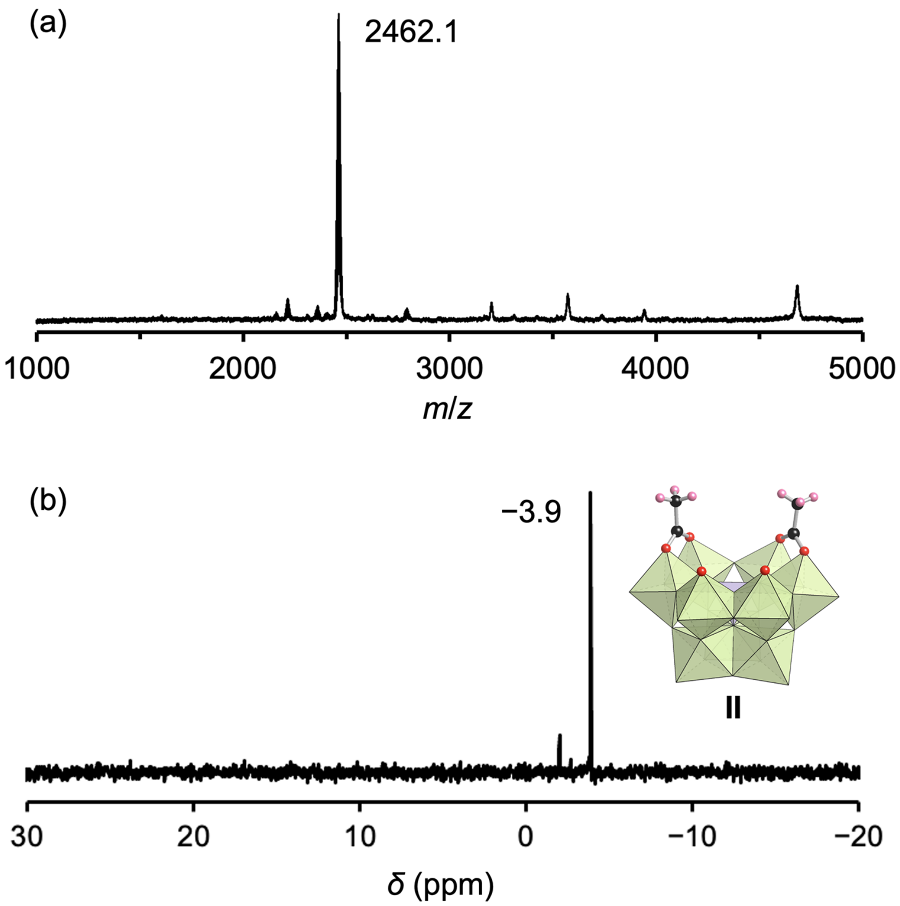

Fig. S1 (a) Positive-ion CSI mass spectrum of the synthetic solution of **II** (diluted by acetonitrile for the mass measurement). A set of signals centered at *m*/*z* 2462.1 can be assigned to $[TBA_4(PMo_9O_{30})(CH_3COO)_2]^+$ (theoretical *m*/*z*: 2462.5). (b) ^{31}P NMR spectrum of the synthetic solution of **II** in acetic anhydride.

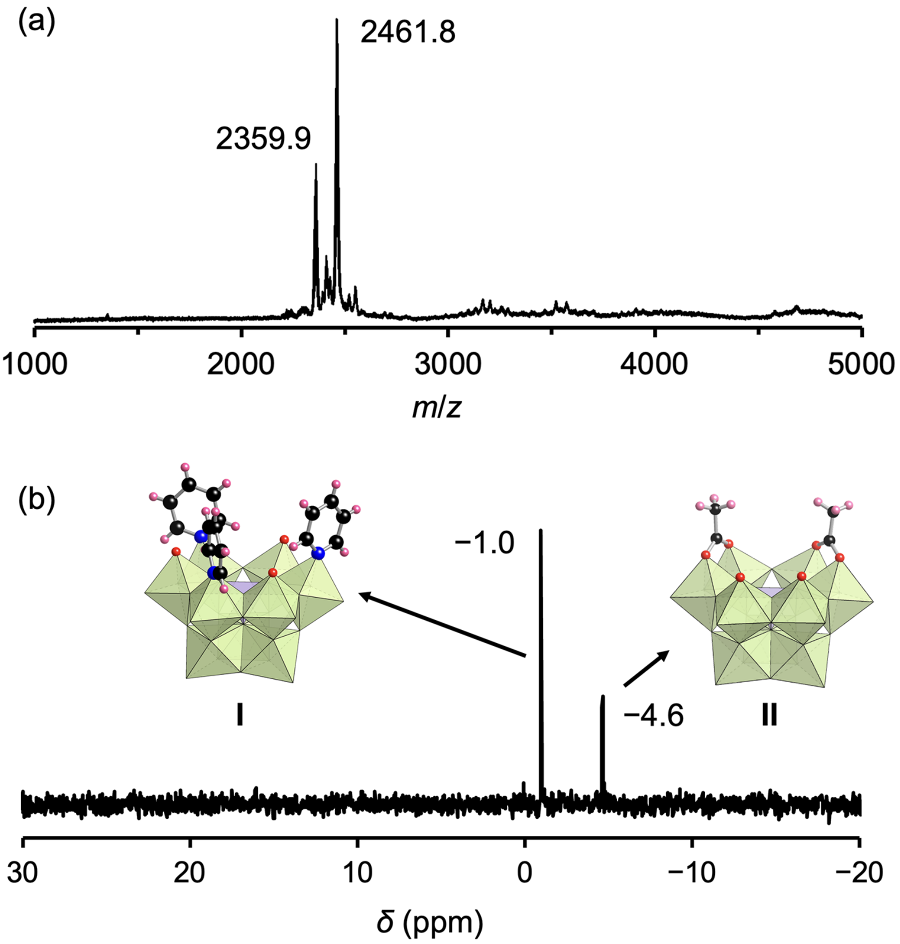

Fig. S2 (a) Positive-ion CSI mass spectrum of the reaction solution of **I** and 1.5 equivalents of acetic anhydride in 1,2-dichloroethane (diluted by acetonitrile for the mass measurement). Sets of signals centered at *m*/*z* 2359.9 and 2461.8 can be assigned to $[TBA_4(PMo_9O_{31})]^+$ (theoretical *m*/*z*: 2360.5) and $[TBA_4(PMo_9O_{30})(CH_3COO)_2]^+$ (theoretical *m*/*z*: 2462.5), respectively. (b) ^{31}P NMR spectra of the reaction solution of **I** and 1.5 equivalents of acetic anhydride in 1,2-dichloroethane.

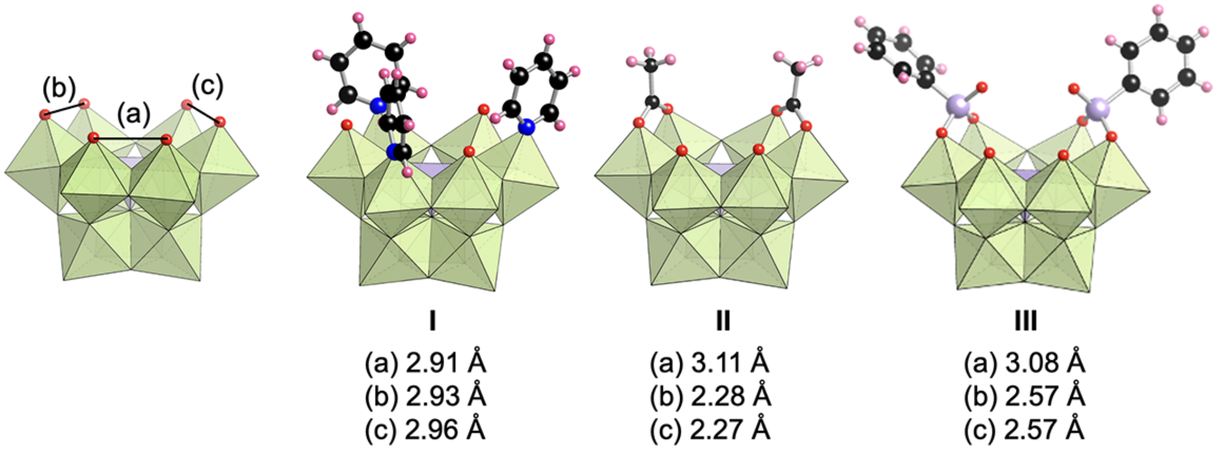

Fig. S3 The distances between adjacent atoms (O or N) at the vacant sites of **I**, **II**, and **III**.

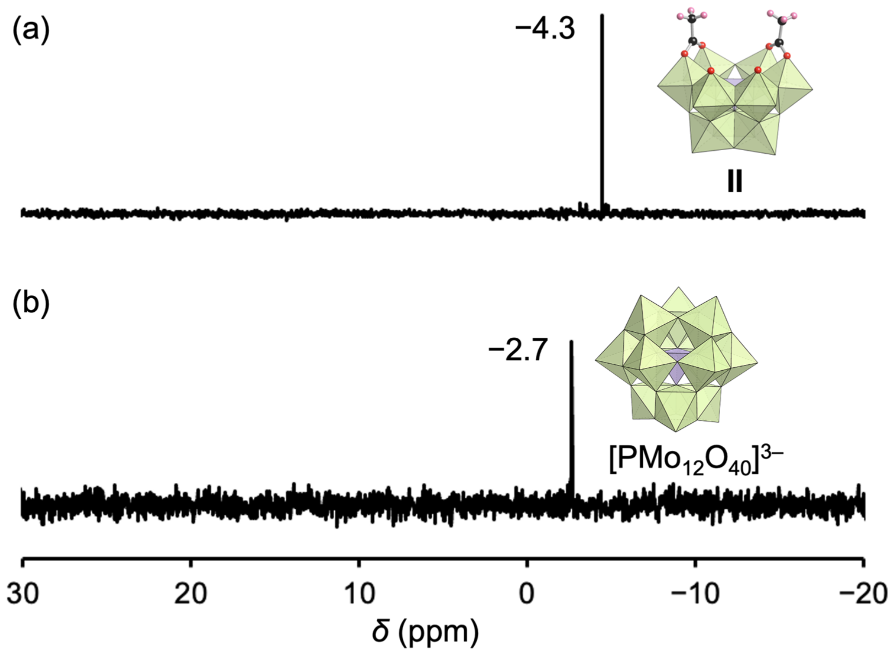

Fig. S4 ^{31}P NMR spectra of **II** (a) just after dissolution and (b) after five days in acetonitrile-d_3.

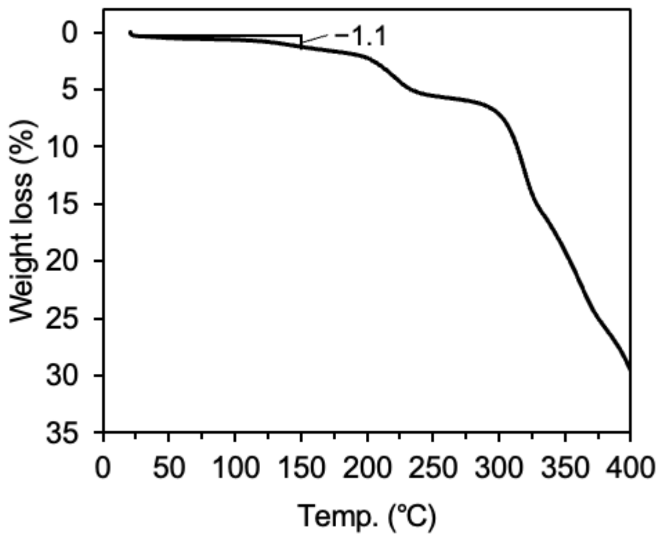

Fig. S5 TG curve of **II** (N_2 atmosphere). The weight loss of 1.1 wt% supported the presence of one water molecule in **II** ($TBA_3[PMo_9O_{30}(CH_3COO)_2](H_2O)$).

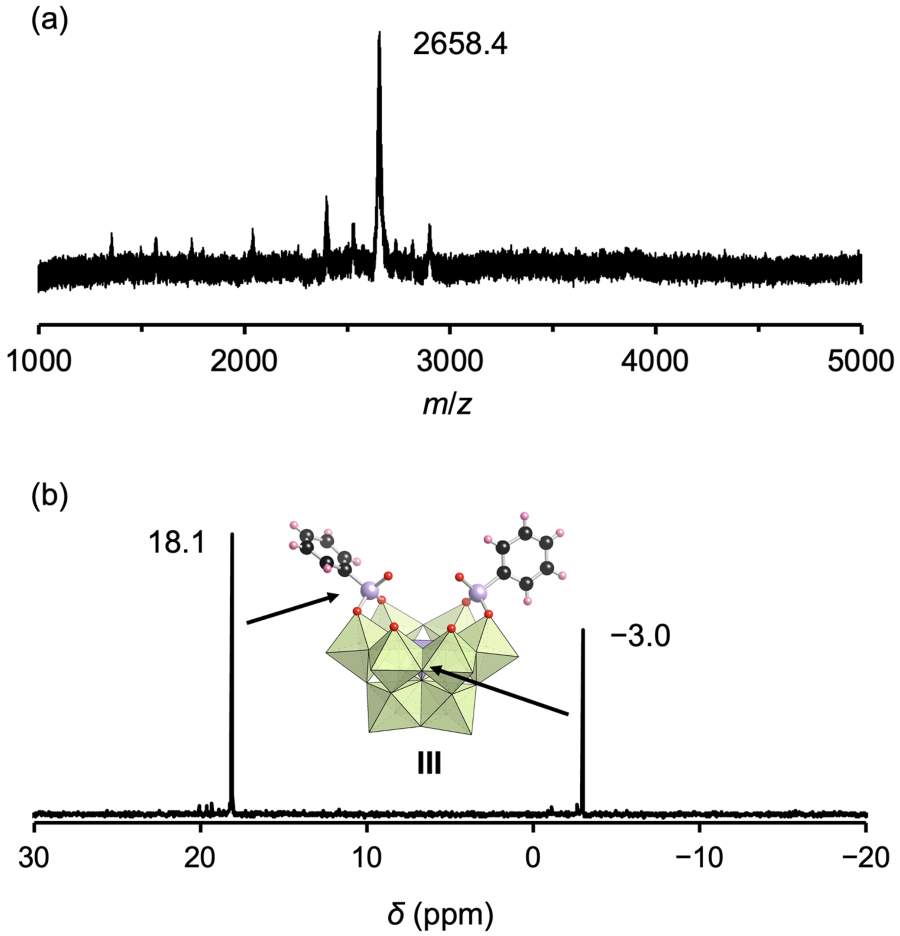

Fig. S6 (a) Positive-ion CSI mass spectrum of the synthetic solution of **III** (diluted by acetonitrile for the mass measurement). A set of signals centered at *m*/*z* 2658.4 can be assigned to $[TBA_4H_2(PMo_9O_{30})(C_6H_5PO_3)_2]^+$ (theoretical *m*/*z*: 2658.6). (b) ^{31}P NMR spectrum of the synthetic solution of **III** in nitromethane.

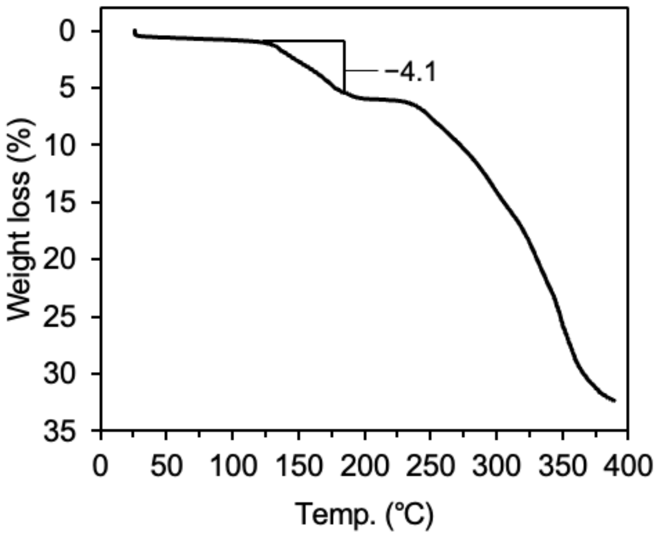

Fig. S7 TG curve of **III** (N_2 atmosphere). The weight loss of 4.1 wt% supported the presence of solvent molecules in **III** ($TBA_3H_2[PMo_9O_{30}(C_6H_5PO_3)_2](H_2O)(C_5H_5N)_{0.5}(CH_3NO_2)_{0.5}$).

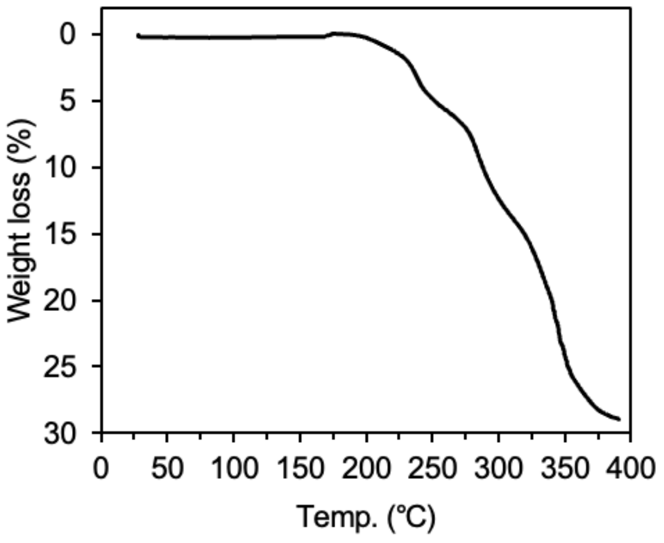

Fig. S8 TG curve of **III$_{Cu}$** (N_2 atmosphere).

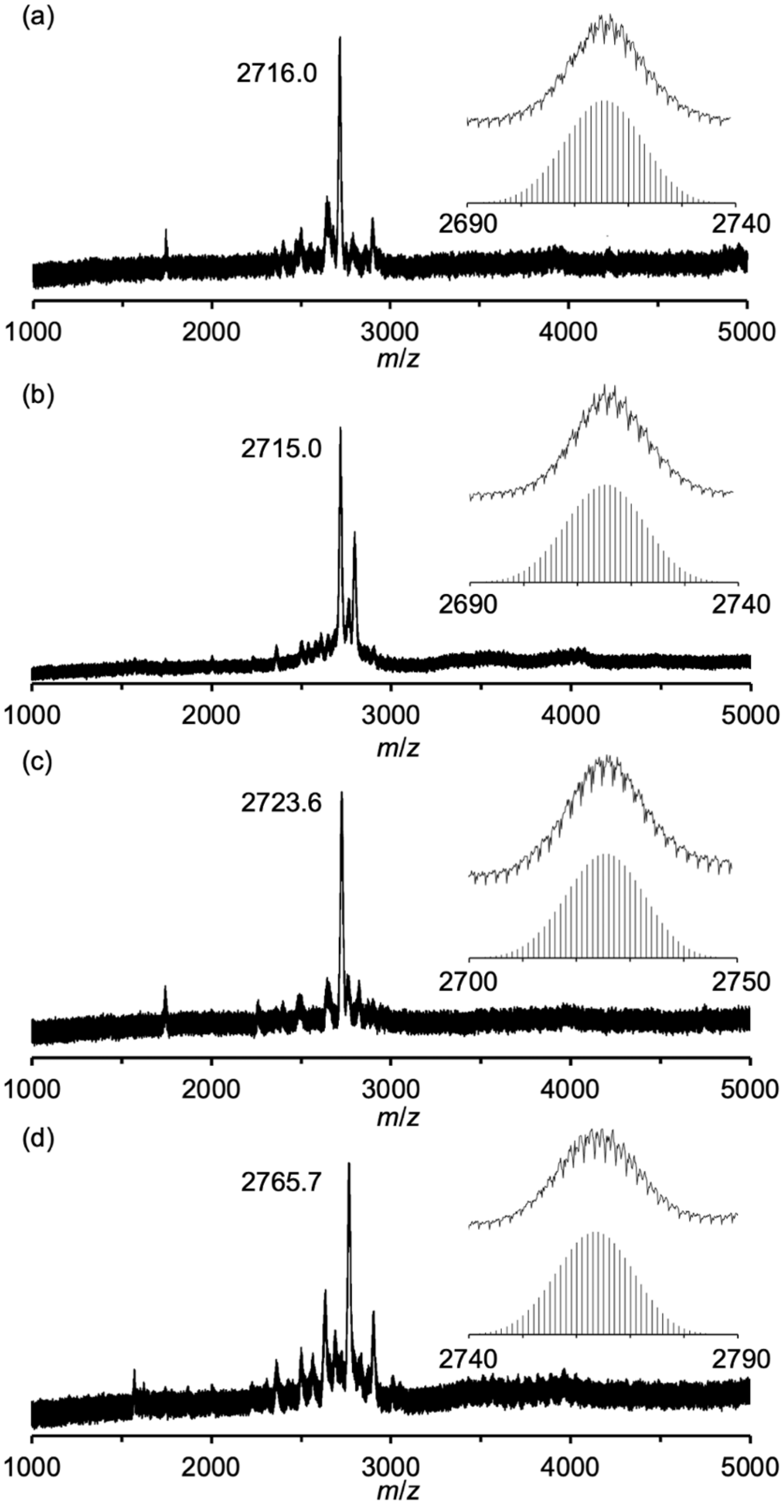

Fig. S9 Positive-ion CSI mass spectra of the reaction solution of **III** with (a) cobalt(II) acetylacetonate dihydrate, (b) nickel(II) acetylacetonate dihydrate, (c) vanadyl acetylacetonate, and (d) silver acetate (diluted by acetonitrile for the mass measurement). Inset: enlarged spectra and simulated patterns for (a) $[TBA_4(PMo_9O_{30})(C_6H_5PO_3)_2Co]^+$ (theoretical *m*/*z*: 2715.5), (b) $[TBA_4(PMo_9O_{30})(C_6H_5PO_3)_2Ni]^+$ (theoretical *m*/*z*: 2715.3), (c) $[TBA_4(PMo_9O_{30})(C_6H_5PO_3)_2VO]^+$ (theoretical *m*/*z*: 2723.5) and (d) $[TBA_4H(PMo_9O_{30})(C_6H_5PO_3)_2Ag]^+$ (theoretical *m*/*z*: 2765.5).

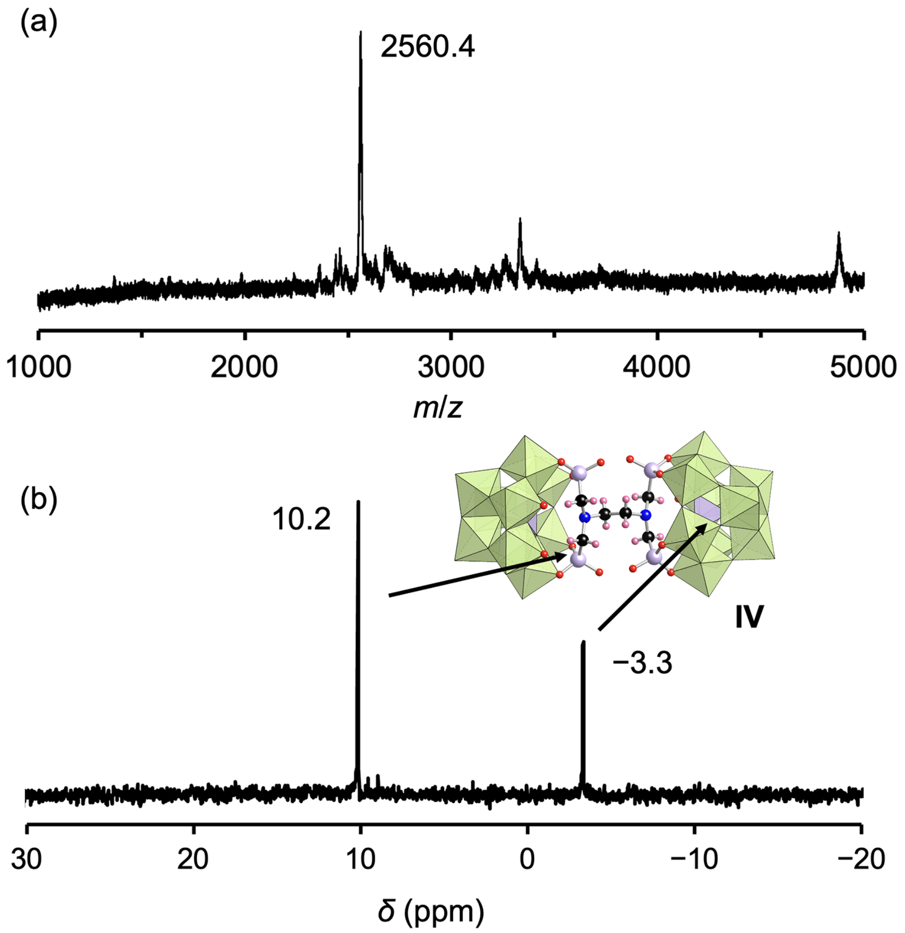

Fig. S10 (a) Positive-ion CSI mass spectrum of the synthetic solution of **IV** (diluted by acetonitrile for the mass measurement). A set of signals centered at *m*/*z* 2560.4 can be assigned to $[TBA_8H_4(PMo_9O_{30})_2(C_6H_{12}N_2P_4O_{12})_2]^{2+}$ (theoretical *m*/*z*: 2560.5). (b) ^{31}P NMR spectrum of **IV** in acetonitrile-d_3.

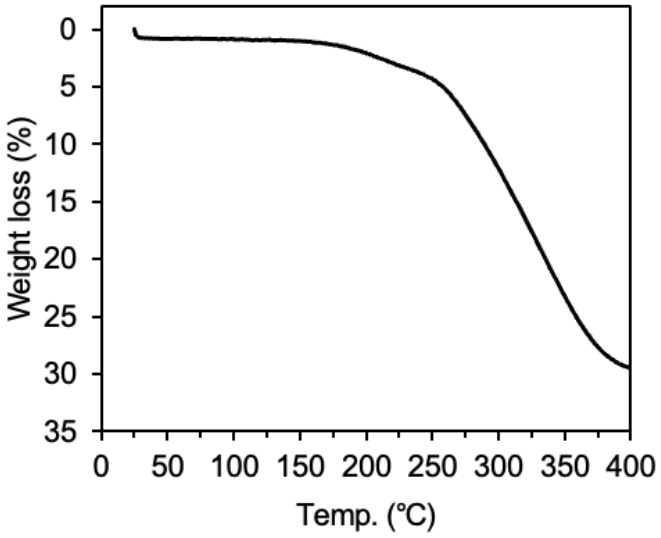

Fig. S11 TG curve of **IV** (N_2 atmosphere).

Additional references

S1 C. Li, N. Mizuno, K. Yamaguchi and K. Suzuki, *J. Am. Chem. Soc.*, 2019, **141**, 7687.

S2 Rigaku OD. CrysAlis PRO. Rigaku Oxford Diffraction Ltd, Yarnton, England (2018).

S3 L. J. Farrugia, *J. Appl. Cryst.*, 1999, **32**, 837.

S4 (a) G. M. Sheldrick, *Acta Cryst.*, 2008, **A64**, 112; (b) G. M. Sheldrick, *Acta Cryst.*, 2015, **C71**, 3.

S5 P. van der Sluis, A. L. Spek, *Acta Cryst.*, 1990, **A46**, 194.

S6 N. E. Brese and M. O'Keeffe, *Acta Cryst.*, 1991, **B47**, 192.

S7 I. D. Brown and D. Altermatt, *Acta Cryst.*, 1985, **B41**, 244.
